# Inflammatory Biomarkers and Left Atrial Remodelling for Early Risk Stratification of 6-Month Readmission in Ischaemic and Idiopathic Dilated Cardiomyopathy

**DOI:** 10.3390/medicina62101884

**Published:** 2026-09-29

**Authors:** Cristian-Viorel Vladutu, Eugen-Nicolae Tieranu, Alexandru Rocsoreanu, Cristian Militaru, Sebastian Militaru, Carmen-Daniela Neagoe

**Affiliations:** 1 Faculty of Medicine, University of Medicine and Pharmacy of Craiova, Petru Rareş Stret 2, 200349 Craiova, Romania; viorel.vladutu@umfcv.ro (C.-V.V.); alexandru.rocsoreanu@umfcv.ro (A.R.); crmilitaru@gmail.com (C.M.); sebastian.militaru@umfcv.ro (S.M.); daniela.neagoe@umfcv.ro (C.-D.N.); 2 Craiova County Emergency Clinical Hospital, Tabaci Street 1, 200642 Craiova, Romania; 3 Research Center of Gastroenterology and Hepatology, University of Medicine and Pharmacy of Craiova, 1 Mai Boulevard 66, 200638 Craiova, Romania

**Keywords:** heart failure, C-reactive protein, fibrinogen, hypertension, dyslipidemia, left atrial volume index, NT-proBNP, readmission, risk stratification, cardiomyopathy

## Abstract

*Background and Objectives*: Inflammatory activation and left atrial remodelling may capture complementary dimensions of residual risk in heart failure with reduced ejection fraction (HFrEF). This study evaluated the prognostic value of routinely available inflammatory biomarkers and the left atrial volume index (LAVI) for early identification of patients at risk of heart-failure-related readmission. *Materials and Methods*: In this single-centre observational cohort study, 150 clinically stable patients with HFrEF were enrolled, including 75 with ischaemic cardiomyopathy and 75 with idiopathic dilated cardiomyopathy. Baseline C-reactive protein (CRP), fibrinogen, neutrophil-to-lymphocyte ratio (NLR), N-terminal pro-B-type natriuretic peptide (NT-proBNP), and LAVI were assessed. The primary outcome was documented readmission for heart-failure decompensation within 6 months. Group comparisons, Spearman correlations, logistic regression, receiver-operating-characteristic analysis, and bootstrap internal validation were performed. *Results*: All participants completed 6-month follow-up, no deaths occurred, and 45 patients (30.0%) were readmitted. Compared with non-readmitted patients, those readmitted had higher NT-proBNP (3058 (2019–4567) vs. 648 (229–1267) pg/mL), fibrinogen (407.8 ± 59.0 vs. 331.3 ± 52.3 mg/dL), CRP (5.60 (3.86–7.82) vs. 2.12 (1.00–2.84) mg/L), and LAVI (33.90 (32.70–34.50) vs. 30.90 (30.20–31.90) mL/m^2^; all *q* < 0.001). In the primary model, each CRP doubling (adjusted odds ratio (OR) 16.16, 95% confidence interval (CI) 4.62–56.51) and each 1 mL/m^2^ increase in LAVI (OR 2.50, 95% CI 1.23–5.10) independently predicted readmission. The augmented model achieved an apparent AUC of 0.962 and a bootstrap-corrected AUC of 0.944, compared to 0.856 and 0.828, respectively, for the clinical model. *Conclusions*: Adding CRP and LAVI to a conventional clinical model provided substantial incremental prognostic information for 6-month readmission in HFrEF. The findings support the integration of inflammatory and left-atrial remodelling measures with, rather than in place of, established clinical risk factors, although external validation is required before clinical implementation.

## 1. Introduction

Heart failure (HF) is a complex clinical syndrome characterized by symptoms and signs resulting from structural or functional cardiac abnormalities and corroborated by elevated natriuretic peptide concentrations or objective evidence of pulmonary or systemic congestion [1]. Heart failure with reduced ejection fraction (HFrEF), conventionally defined by a left ventricular ejection fraction of ≤40%, remains associated with substantial mortality, recurrent hospitalization, impaired functional capacity, and poor health-related quality of life [1,2,3,4,5], although the implementation of comprehensive guideline-directed medical therapy—including renin–angiotensin system inhibition or angiotensin receptor–neprilysin inhibition, evidence-based beta-blockade, mineralocorticoid receptor antagonism, and sodium–glucose cotransporter 2 inhibition—has markedly improved clinical outcomes, considerable residual risk persists [2,3,5]. This residual risk reflects the biological heterogeneity of HFrEF and underscores the need for biomarkers capable of characterizing complementary pathophysiological domains beyond conventional measures of cardiac structure and function.

Circulating biomarkers have become integral to the diagnosis, risk stratification, and longitudinal assessment of patients with HF [6]. Among them, N-terminal pro-B-type natriuretic peptide (NT-proBNP) is the most extensively validated. NT-proBNP is released predominantly by ventricular cardiomyocytes in response to increased myocardial wall stress and is influenced by intracardiac filling pressures, ventricular dilatation, myocardial ischemia, neurohormonal activation, and adverse cardiac remodelling [6]. Its relatively long plasma half-life and analytical stability make it particularly suitable for clinical assessment. Nevertheless, NT-proBNP is not a disease-specific marker, and its concentration is affected by age, renal function, body mass index, cardiac rhythm, sex, pulmonary vascular disease, and several extracardiac comorbidities [6]. Consequently, NT-proBNP should be interpreted as an integrated marker of hemodynamic and myocardial stress rather than a direct measurement of one isolated biological process.

Inflammation represents another fundamental component of HFrEF pathophysiology. Both experimental and clinical evidence indicate that myocardial injury and persistent hemodynamic overload activate innate and adaptive immune pathways, leading to leukocyte recruitment, cytokine release, endothelial dysfunction, oxidative stress, extracellular-matrix turnover, cardiomyocyte dysfunction, and progressive myocardial fibrosis [7,8,9,10,11,12,13]. Initially adaptive inflammatory signalling may become maladaptive when resolution pathways are insufficient, thereby perpetuating adverse ventricular remodelling and systemic organ dysfunction [7,8,9,13]. In addition to myocardial inflammation, venous congestion, intestinal barrier dysfunction, endotoxin translocation, adipose-tissue inflammation, renal dysfunction, and associated metabolic disease may sustain a systemic inflammatory state in patients with chronic HF [7,8,10,11,12]. The inflammatory phenotype of HFrEF is therefore unlikely to be captured by a single biomarker, and clinically accessible markers may reflect distinct but overlapping components of immune activation, acute-phase signalling, coagulation, and cellular stress.

NT-proBNP concentrations provide powerful prognostic information across the spectrum of HFrEF. However, randomized evidence has not demonstrated that adjustment of therapy to achieve predefined natriuretic peptide targets is superior to high-quality guideline-directed clinical care [14]. Conversely, reductions in NT-proBNP during follow-up are consistently associated with reverse cardiac remodelling and improved outcomes [15,16,17]. Serial NT-proBNP measurements may provide incremental prognostic information, particularly when interpreted in relation to treatment implementation and disease trajectory [18,19,20]. Important biological modifiers must nevertheless be considered. Obesity is associated with lower natriuretic peptide concentrations for a given degree of hemodynamic impairment, while atrial fibrillation and renal dysfunction may produce disproportionately elevated values [19,20,21,22]. NT-proBNP also identifies high-risk populations and treatment-responsive phenotypes in contemporary HFrEF trials, including studies of vericiguat, dapagliflozin, and empagliflozin [23,24,25,26,27,28]. However, marked residual risk may persist despite reductions in NT-proBNP and optimization of guideline-directed therapy [15,18,29], suggesting that hemodynamic improvement does not necessarily indicate complete resolution of systemic inflammatory and metabolic abnormalities.

C-reactive protein (CRP), particularly when measured using a high-sensitivity assay, is one of the most widely investigated inflammatory biomarkers in HF. CRP is predominantly synthesized by hepatocytes under stimulation from interleukin-6 and related cytokines and reflects activation of the systemic acute-phase response. Elevated CRP concentrations have been associated with impaired exercise capacity, greater symptom burden, congestion, adverse ventricular remodelling, recurrent HF hospitalization, and mortality [30,31,32,33,34,35,36,37,38,39]. Importantly, CRP and NT-proBNP may convey complementary rather than interchangeable information. In patients with HFrEF, both biomarkers have been independently associated with functional limitation, whereas their direct correlation has been modest or absent in some cohorts [30]. This observation supports the concept that myocardial wall stress and systemic inflammation represent partially dissociable biological processes. CRP may also identify a subgroup of patients with a persistent inflammatory phenotype despite otherwise optimized pharmacological treatment [31]. In chronic and recently worsening HFrEF, higher high-sensitivity CRP has been associated with an increased incidence of cardiovascular death and HF hospitalization [32,33]. Similar associations have been observed in acute HF and population-based studies of incident HF [34,35]. Furthermore, longitudinal or cumulative exposure to elevated CRP may provide information beyond a single baseline measurement [36,37]. Meta-analytic evidence supports an association between increased CRP and adverse HF outcomes, although heterogeneity in study populations, assay methods, clinical settings, and covariate adjustment remains substantial [38]. Data from HF with preserved ejection fraction similarly suggest that inflammation defines clinically relevant phenotypes, but extrapolation to HFrEF should remain cautious because of differences in pathophysiology and comorbidity burden [39,40]. Moreover, the variable results of anti-inflammatory interventions in systolic HF indicate that elevated CRP may function as both a risk marker and a correlate of disease severity, without necessarily identifying a uniformly modifiable causal pathway [41].

Fibrinogen provides a potential link between systemic inflammation, coagulation, endothelial dysfunction and cardiovascular remodelling. As a positive acute-phase protein synthesized by the liver, fibrinogen increases in response to inflammatory cytokines. In addition to its central role in clot formation and platelet aggregation, elevated fibrinogen may increase blood viscosity, promote endothelial activation, facilitate leukocyte adhesion, and contribute to a prothrombotic milieu. In patients with HF, higher fibrinogen concentrations have been associated with disease severity and adverse cardiovascular outcomes [42]. More recently, the fibrinogen-to-albumin ratio has emerged as an integrated inflammatory and nutritional index associated with mortality, rehospitalization, and worse clinical status in both acute and chronic HF populations [43,44,45,46]. Some studies have reported associations between the fibrinogen-to-albumin ratio and NT-proBNP concentrations, suggesting a relationship between inflammatory-thrombotic activation and hemodynamic stress [43,44,45]. Nevertheless, evidence specifically examining the correlation between absolute fibrinogen and NT-proBNP in well-characterized HFrEF populations remains limited. Furthermore, albumin-dependent indices may be influenced by hepatic congestion, renal protein loss, malnutrition, and systemic illness, making it difficult to determine the independent contribution of fibrinogen. The established role of fibrinogen in atherothrombosis and vascular inflammation further supports its potential relevance in ischemic and nonischemic HFrEF, while also emphasizing the need to account for underlying HF etiology [47].

The neutrophil-to-lymphocyte ratio (NLR), calculated from a routine complete blood count, is an inexpensive and readily available marker integrating two complementary components of the leukocyte response. Neutrophilia may reflect innate immune activation, catecholamine- and cortisol-mediated stress, oxidative injury, tissue congestion, or occult infection, whereas relative lymphopenia may reflect impaired adaptive immunity and increased neuroendocrine stress. A meta-analysis demonstrated that elevated NLR is associated with increased mortality and adverse outcomes in HF [48]. Subsequent studies have linked higher NLR with disease severity, reduced functional capacity, rehospitalization, and both short- and long-term mortality [49,50,51,52]. NLR has also been incorporated into composite indices designed to improve characterization of inflammatory and nutritional risk [53]. Contemporary reviews emphasize its potential clinical usefulness while acknowledging the absence of standardized cut-off values and its sensitivity to infection, corticosteroid treatment, hematological disorders, malignancy, smoking, and other inflammatory comorbidities [54]. Of particular relevance, NLR has demonstrated prognostic value comparable or complementary to NT-proBNP in some HF cohorts, and significant associations between the two markers have been reported [50,55]. More recent HFrEF-specific observations continue to support NLR as an accessible indicator of residual inflammatory risk [56]. However, differences in clinical setting, HF phenotype, timing of blood sampling, and adjustment for confounding factors have limited the generalizability of available findings.

The simultaneous evaluation of CRP, fibrinogen, and NLR may provide a broader representation of the inflammatory milieu than assessment of any single marker. CRP primarily reflects cytokine-mediated hepatic acute-phase activity; fibrinogen reflects both inflammation and thrombotic–hemorheological activation; and NLR reflects the balance between innate cellular activation and adaptive immune competence. Examining their relationships with NT-proBNP could therefore help determine whether the magnitude of systemic inflammation parallels the degree of myocardial wall stress in HFrEF or whether inflammatory activation identifies an additional biological axis that is incompletely captured by natriuretic peptides. Such an approach may be particularly relevant because apparently similar NT-proBNP concentrations can occur in patients with substantially different comorbidity profiles, immune activation, congestion patterns, and clinical trajectories.

Current evidence supports the individual prognostic value of NT-proBNP, CRP, fibrinogen-related indices, and NLR, but direct comparative analyses of their interrelationships in HFrEF remain comparatively scarce [30,42,43,44,45,46,48,49,50,51,52,53,54,55,56]. Available findings are also potentially affected by age, sex, obesity, renal dysfunction, atrial fibrillation, ischemic etiology, diabetes mellitus, anemia, infection, functional status, congestion, and background HF therapy. A carefully adjusted analysis is therefore required before any observed association can be interpreted as evidence of pathophysiological coupling. Correlation itself cannot establish causality; nevertheless, it may generate clinically relevant hypotheses concerning the coexistence of hemodynamic stress and systemic inflammatory activation.

Accordingly, the present study aimed to determine whether routinely available inflammatory biomarkers—CRP, fibrinogen, and NLR—and LAVI could improve early risk stratification for heart-failure-related readmission within 6 months in patients with HFrEF due to ischaemic cardiomyopathy or idiopathic dilated cardiomyopathy. The analytical objectives were to compare biomarker and structural profiles according to readmission status and cardiomyopathy etiology; quantify their relationships with NT-proBNP; identify independent predictors of readmission using multivariable regression; and evaluate discrimination, clinically relevant thresholds, and internal model validity. We hypothesized that systemic inflammatory activation and left atrial remodelling would provide complementary prognostic information beyond conventional clinical characteristics.

## 2. Materials and Methods

### 2.1. Study Design and Setting

This was a single-centre observational cohort study with prospective 6-month follow-up. The analytical cohort comprised 150 adult patients with chronic HFrEF who were evaluated in the Cardiology Department of the Craiova County Emergency Clinical Hospital during an enrolment period from October 2024 through January 2026. Eligible patients were enrolled consecutively. The ischaemic cardiomyopathy cohort reached 75 participants first; thereafter, enrolment continued consecutively among eligible patients with idiopathic dilated cardiomyopathy until the same number (*n* = 75) was reached. Thus, the equal group sizes resulted from sequential completion of the two consecutively enrolled aetiological cohorts rather than from matching or post hoc selection. Recruitment of the two cohorts was completed only one month apart. The prospective 6-month follow-up was completed in July 2026. HFrEF was defined by a left ventricular ejection fraction (LVEF) < 40% measured by transthoracic echocardiography using the biplane Simpson method. Patients were clinically stable at enrolment and were admitted for diagnostic evaluation or scheduled interventional procedures rather than for acute coronary syndrome or acute heart-failure decompensation.

### 2.2. Eligibility Criteria and Cardiomyopathy Classification

Patients were eligible for enrolment if they fulfilled all of the following criteria: (i) established chronic HFrEF with an LVEF < 40% documented by transthoracic echocardiography using the biplane Simpson method; (ii) an underlying diagnosis of ischaemic cardiomyopathy or idiopathic dilated cardiomyopathy; (iii) clinical stability at baseline, defined by the absence of acute coronary syndrome and heart-failure decompensation during the preceding 6 months; (iv) admission for scheduled diagnostic assessment, clinical reassessment, or an elective interventional procedure rather than for an acute cardiovascular event; and (v) provision of written informed consent. Eligibility was not restricted by sex, place of residence, or NYHA functional class.

Exclusion criteria were conditions likely to confound the interpretation of circulating inflammatory biomarkers or to indicate recent clinical instability. Patients were excluded if they had an active bacterial, fungal, or viral infection; a known autoimmune or other systemic inflammatory disease; active malignancy; or clinical signs or symptoms at enrolment suggesting an active inflammatory disorder. Additional exclusion criteria were acute coronary syndrome within the previous 6 months and heart-failure decompensation within the previous 6 months. These criteria were applied before laboratory biomarker assessment and study-group allocation.

The cohort was classified into two aetiological groups of equal size according to the enrolment scheme described above. Ischaemic cardiomyopathy was established by prior coronary angiography demonstrating significant coronary artery disease, defined as >50% stenosis of the left main coronary artery or >70% stenosis of another major epicardial coronary artery, together with clinical, electrocardiographic, and echocardiographic evidence compatible with previous myocardial infarction. The non-ischaemic group consisted exclusively of patients with idiopathic dilated cardiomyopathy, diagnosed after exclusion of obstructive coronary disease and relevant valvular causes. Cardiac magnetic resonance imaging, including the distribution and pattern of late gadolinium enhancement (LGE), contributed to the aetiological classification by supporting a non-ischaemic phenotype and excluding an ischaemic scar substrate. Other acquired causes of dilated cardiomyopathy, including alcohol-related cardiomyopathy, a history compatible with prior myocarditis, tachyarrhythmia-mediated cardiomyopathy, and chemotherapy-related myocardial injury, were excluded through clinical history and review of the patients’ available medical records.

### 2.3. Clinical and Laboratory Assessment

Baseline demographic and clinical variables included age, sex, New York Heart Association (NYHA) functional class, obesity, current smoking, hypertension, dyslipidaemia, chronic kidney disease stage 3 or higher, anemia, and atrial fibrillation. Diagnoses of obesity, anemia, chronic kidney disease, hypertension, and dyslipidaemia were established according to the guideline-based diagnostic criteria applicable during the enrolment period and were recorded as present or absent in the analytical database. Obesity was defined according to the anthropometric criteria used in the study protocol, including body mass index > 30 kg/m^2^ and sex-specific central-adiposity thresholds. Anemia was classified using the lower laboratory hemoglobin reference limit of 11.5 g/dL. All categorical variables were coded as present or absent in the analytical database.

Approximately 15 mL of venous blood was collected at baseline under standardized pre-analytical conditions. The analyzed variables included NT-proBNP, standard CRP, fibrinogen, and the NLR calculated from the complete blood count. NT-proBNP was quantified using an automated electrochemiluminescence immunoassay (ECLIA), in accordance with the reagent manufacturer’s analytical instructions and the laboratory quality-control procedures. In the sandwich ECLIA principle, NT-proBNP in the patient sample binds to two specific monoclonal antibodies: a biotinylated capture antibody and a second antibody labelled with an electrochemiluminescent ruthenium complex. The resulting immunocomplexes bind to streptavidin-coated microparticles and are magnetically captured on the electrode surface of the measuring cell. After removal of unbound material, application of an electrical potential induces light emission from the ruthenium label; the emitted signal is measured photometrically and converted to NT-proBNP concentration using the assay calibration curve. The local laboratory reference interval for NT-proBNP was 0–125 pg/mL, and concentrations > 125 pg/mL were classified as elevated. Standard CRP was measured with the Tina-quant C-Reactive Protein IV (CRP4) reagent (Roche Diagnostics) on a cobas c 501 analyser, using a latex-enhanced immunoturbidimetric method. The adult reference value was <5 mg/L; for the prespecified categorical analysis, CRP > 5 mg/L was classified as elevated. Fibrinogen samples were collected in tubes containing 3.2% sodium citrate. The local laboratory reference interval for fibrinogen was 238–498 mg/dL; fibrinogen < 238 mg/dL was classified as below the reference interval and fibrinogen > 498 mg/dL as elevated.

### 2.4. Echocardiographic Assessment

Two-dimensional transthoracic echocardiography was performed using GE Vivid S80 and Philips Affiniti CVx systems with dedicated cardiac transducers. Participants were examined in the left lateral decubitus position. Standard views were optimized for each patient, and echocardiographic acquisition and measurements were performed in accordance with European Association of Cardiovascular Imaging recommendations for transthoracic echocardiography. Left atrial volume was assessed by two-dimensional echocardiography from dedicated left-atrial apical four-chamber and two-chamber views, with particular care to avoid foreshortening. Maximum left atrial volume (LAVmax) was measured at the end of ventricular systole, immediately before mitral-valve opening, by tracing the left-atrial endocardial border in both apical views. Pulmonary veins and the left atrial appendage were excluded from the contour, which was closed at the level of the mitral annulus. LAVmax was calculated using the biplane modified Simpson method and then indexed to body surface area to derive LAVI. In patients in sinus rhythm, three separate measurements were obtained under the same standardized acquisition protocol and averaged for analysis. In patients with atrial fibrillation, LAVmax measurements from five cardiac cycles were averaged before indexing to body surface area. LAVI < 34 mL/m^2^ was considered normal; therefore, LAVI ≥ 34 mL/m^2^ defined left atrial enlargement. Echocardiographers were unaware of the inflammatory-biomarker results at the time of image acquisition and measurement.

### 2.5. Outcome Definition and Follow-Up

The primary outcome was at least one documented hospital readmission for heart-failure decompensation within 6 months after enrolment. For each participant, the 6-month follow-up period began on the individual date of enrolment in the study. A qualifying readmission was caused by clinically relevant worsening of heart-failure signs and symptoms together with deterioration in NYHA functional class that required hospital assessment and treatment. Endpoint classification was based on the clinical presentation and hospital documentation and was determined independently of baseline biomarker concentrations; baseline biomarker values were not used to define whether a readmission met the study endpoint. Throughout the study period, all patients were managed according to the contemporary European Society of Cardiology recommendations for heart failure applicable during the study period. Guideline-directed medical therapy was implemented using the foundational treatment pillars whenever tolerated, with treatment initiation, adjustment, and up-titration individualized to the maximally tolerated regimen for each patient. Because the analytical database was designed primarily for prognostic biomarker and clinical-variable assessment, it did not record patient-level details for each heart-failure medication or the exact dose received by each participant. Follow-up status was established by direct patient contact and verified using supporting hospital documentation. Vital status was also ascertained at 6 months; all 150 participants completed follow-up and no deaths occurred. The analytical database recorded readmission as a binary outcome and did not include the exact date of readmission or the number of recurrent admissions. Consequently, survival, competing-risk, and recurrent-event models were not applicable, and the prognostic analyses focused on the occurrence of at least one readmission during the individual 6-month follow-up period.

### 2.6. Data Management and Statistical Analysis Using Microsoft Excel and Python

Data entry, coding, initial quality control, and selected statistical procedures were performed in Microsoft Excel with the Analysis ToolPak enabled, while advanced inferential modelling, resampling, and figure generation were conducted in Python 3.14.7. The database was structured with one participant per row and one variable per column; binary variables were coded as 0/1. Before analysis, both platforms were used to verify variable ranges, coding consistency, missingness, unique patient identifiers, duplicate clinical profiles, and implausible values. All 150 records were complete and unique, so no imputation was required. Derived variables included age/5, fibrinogen/50, log2 CRP [Excel formula: =LOG(cell,2)], log2 NT-proBNP, NT-proBNP > 125 pg/mL, CRP > 5 mg/L, fibrinogen < 238 mg/dL, fibrinogen > 498 mg/dL, and LAVI ≥ 34 mL/m^2^.

Descriptive statistics for approximately symmetric continuous variables were obtained using Data Analysis in Excel and independently verified with NumPy and SciPy; results are reported as mean ± standard deviation (SD). For skewed variables, medians and interquartile ranges (IQRs) were calculated using MEDIAN and QUARTILE.INC in Excel and verified in Python. Distributional form was assessed using histograms, skewness and kurtosis indices, normal quantile plots, and visual inspection. Age and fibrinogen were compared using Welch’s two-sample *t*-test, implemented through the Analysis ToolPak option *t*-Test: Two-Sample Assuming Unequal Variances and confirmed with SciPy. NT-proBNP, CRP, NLR, and LAVI were compared using the Mann–Whitney U test in Python; for spreadsheet verification, pooled observations were ranked using RANK.AVG, rank sums and U statistics were derived, and the resulting two-sided *p*-values were checked against the Python output.

Categorical variables were summarized as number (percentage). Pearson chi-square tests were calculated using contingency tables and CHISQ.TEST in Excel and verified in Python; Fisher’s exact test was used in Python when expected cell counts were sparse. Effect sizes were reported as Hedges’ g for parametric continuous comparisons, rank-biserial correlation for Mann–Whitney comparisons, and odds ratios (ORs) for binary variables. Spearman correlations among age, NT-proBNP, fibrinogen, CRP, NLR, and LAVI were obtained in Excel by applying RANK.AVG to each variable followed by the ToolPak Correlation procedure, and were reproduced directly in SciPy. Within prespecified exploratory comparison families, *p*-values were adjusted using the Benjamini–Hochberg false discovery rate (FDR) procedure. In Excel, ordered *p*-values were multiplied by m/i, capped at 1, and subjected to a reverse cumulative minimum to preserve monotonic *q*-values.

Univariable logistic regression was used to describe unadjusted associations. Because only 45 readmission events were available relative to the number of prespecified multivariable predictors, all multivariable logistic models were re-estimated using Firth’s bias-reduced penalized likelihood, based on the Jeffreys-prior penalty, to reduce small-sample coefficient bias and instability. Continuous predictors were scaled for clinical interpretability: age per 5-year increase, fibrinogen per 50 mg/dL increase, LAVI per 1 mL/m^2^ increase, and NLR per one-unit increase. NT-proBNP and CRP were log2-transformed, so their ORs represent the change in odds associated with a doubling of concentration. Adjusted ORs were obtained by exponentiating the penalized coefficients; 95% CIs were derived from profile penalized likelihood, and *p*-values from penalized likelihood-ratio tests. Three models were analyzed hierarchically: a clinical model including age, sex, cardiomyopathy etiology, NYHA class III, hypertension, and atrial fibrillation; a primary augmented model that retained all six clinical predictors and additionally included log2 CRP and LAVI; and an exploratory multimarker model that retained the full primary model and additionally included fibrinogen. The incremental contribution of CRP and LAVI was evaluated primarily by the paired change in discrimination using stratified bootstrap resampling rather than by an unpenalized nested likelihood-ratio test. NT-proBNP and LAVI were not entered simultaneously because of their strong correlation. Dyslipidaemia was excluded from etiology-adjusted models because it perfectly separated ischaemic from idiopathic cardiomyopathy.

The Firth-penalized multivariable coefficients were estimated in Python using an iterative adjusted-score implementation. Penalized coefficient estimation itself was not reproduced with Excel Solver because the Firth penalty requires iterative recomputation of the Fisher-information determinant.

Discrimination was quantified using the area under the receiver-operating-characteristic curve (AUC). In Python, ROC curves, AUCs, operating characteristics, and paired AUC comparisons were calculated with scikit-learn and stratified bootstrap procedures. In Excel, marker values or predicted probabilities were sorted in descending order; COUNTIFS-derived true-positive, false-positive, true-negative, and false-negative counts were used to calculate sensitivity, specificity, positive predictive value (PPV), negative predictive value (NPV), and Youden’s J, and AUC was verified by trapezoidal integration of sensitivity against 1-specificity. Metrics were calculated at the prespecified laboratory/echocardiographic limits and at exploratory, cohort-derived Youden thresholds; these data-derived thresholds were not considered clinical cut-offs and require external validation. Overall predictive accuracy was summarized with the Brier score. Internal validation used 500 stratified bootstrap resamples to estimate optimism-corrected AUCs, Brier scores, and the calibration slope. For the primary penalized model, calibration-in-the-large was quantified as the calibration intercept by fitting a logistic model with the model logit as an offset, for which the ideal value is 0; the apparent calibration slope was estimated by regressing outcome on the model logit, for which the ideal value is 1. Ninety-five percent CIs were reported for both calibration parameters. Graphical calibration was represented by a logistic recalibration curve with a nonparametric bootstrap pointwise 95% confidence band; quintiles of predicted risk were additionally plotted as mean predicted risk versus observed readmission proportion with Wilson 95% CIs. Because calibration was assessed in the derivation cohort, the curve and parameter estimates were considered apparent calibration. Bootstrap validation was used to quantify internal optimism and stability within the derivation dataset, but was not considered a substitute for validation in an independent external cohort. Etiology-by-biomarker interactions, etiology-specific AUCs, and an exploratory HC3-robust linear model of log2 NT-proBNP were evaluated as sensitivity analyses. All tests were two-sided, with *p* < 0.05 considered statistically significant.

Concordance of descriptive statistics, principal group comparisons, correlation coefficients, transformations, ROC metrics, predicted probabilities from the final penalized coefficients, and threshold classifications was checked across the Excel and Python workflows.

## 3. Results

### 3.1. Cohort Characteristics and Data Quality

The analysis included 150 patients, equally distributed between ischaemic cardiomyopathy (*n* = 75) and idiopathic dilated cardiomyopathy (*n* = 75). The mean age was 60.7 ± 3.0 years, 91 participants (60.7%) were men, and 41 (27.3%) were in NYHA functional class III. The database contained no missing values or duplicate patient profiles. Median NT-proBNP was 1043 (526–2404) pg/mL, mean fibrinogen was 354.3 ± 64.6 mg/dL, median CRP was 2.80 (1.62–4.58) mg/L, median NLR was 2.36 (1.83–2.93), and median LAVI was 31.30 (30.52–33.38) mL/m^2^. NT-proBNP exceeded the local upper reference limit of 125 pg/mL in 142 patients (94.7%). CRP exceeded 5 mg/L in 34 patients (22.7%), fibrinogen was below 238 mg/dL in four (2.7%) and above 498 mg/dL in four (2.7%), and LAVI was ≥34 mL/m^2^ in 25 (16.7%).

### 3.2. Cardiomyopathy Etiology

Most continuous biomarker and echocardiographic variables were similar between aetiological groups. The 6-month readmission rate was 33.3% in idiopathic dilated cardiomyopathy and 26.7% in ischaemic cardiomyopathy (*p* = 0.373). Dyslipidaemia was present in all patients with ischaemic cardiomyopathy compared with 34.7% of those with idiopathic dilated cardiomyopathy (*q* < 0.001), resulting in perfect separation between dyslipidaemia and etiology. Chronic kidney disease stage ≥ 3 was numerically more frequent in ischaemic cardiomyopathy (20.0% vs. 5.3%; nominal *p* = 0.012), although this difference did not remain significant after FDR correction (*q* = 0.124). No other etiology comparison was statistically significant after multiplicity adjustment.

### 3.3. Characteristics Associated with 6-Month Readmission

**Figure 1:**
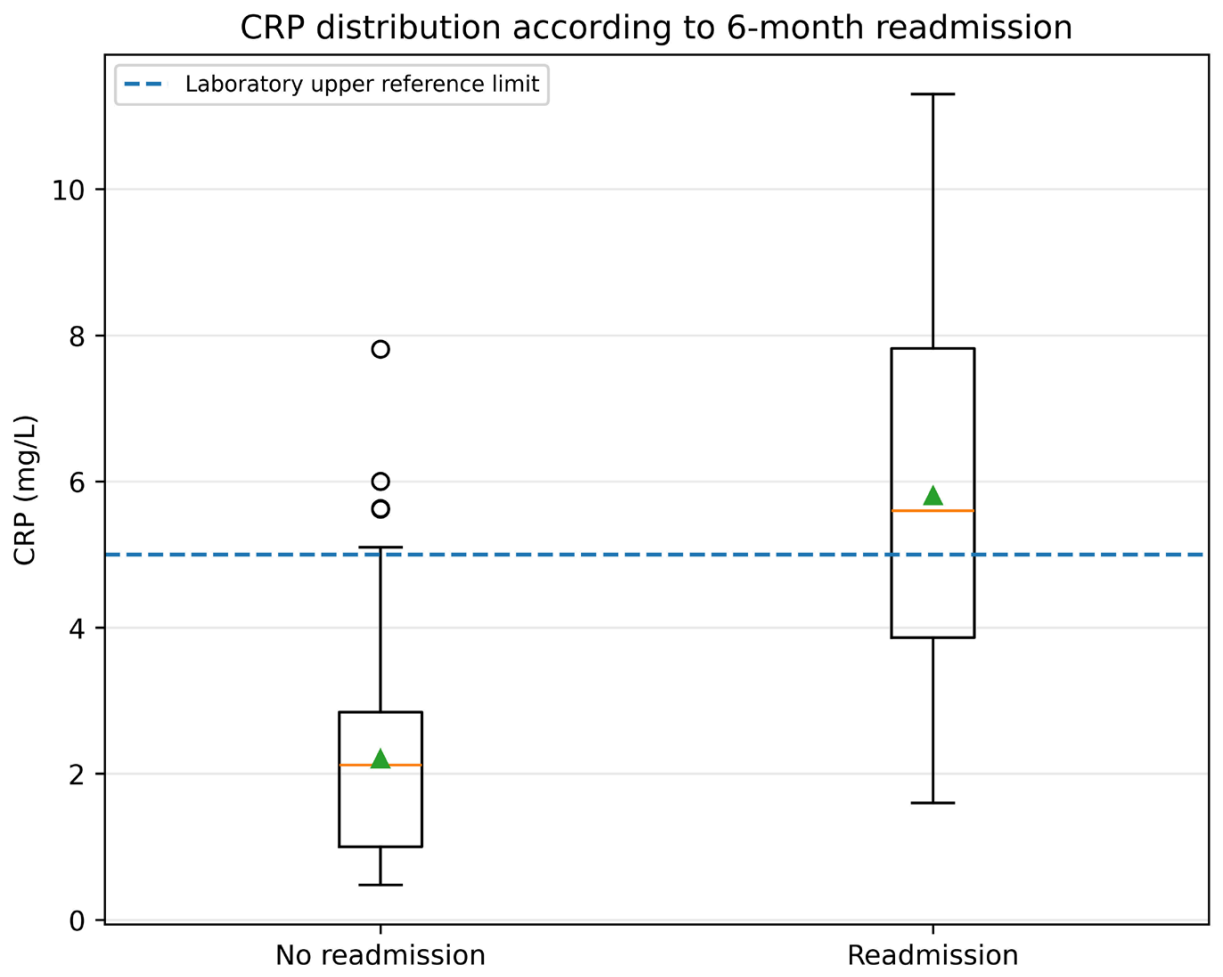
C-reactive protein (CRP) distribution according to 6-month readmission status. Boxes show the interquartile range, centre lines the median, whiskers 1.5 times the interquartile range, circles outliers, and triangles the mean. The dashed line marks the laboratory upper reference limit (5 mg/L). FDR-adjusted *q* < 0.001.

**Figure 2:**
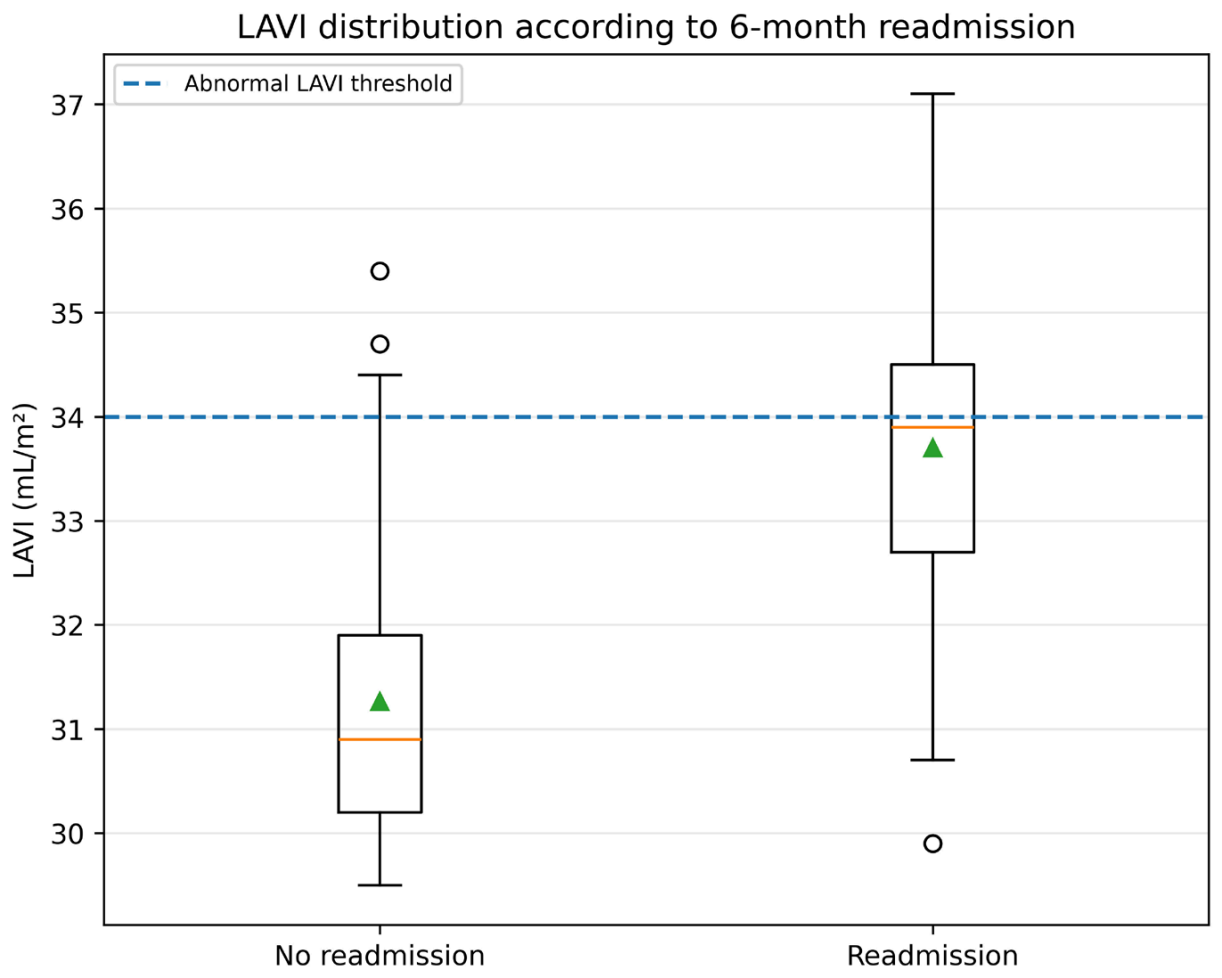
Left atrial volume index (LAVI) distribution according to 6-month readmission status. Boxes show the interquartile range, centre lines the median, whiskers 1.5 times the interquartile range, circles outliers, and triangles the mean. The dashed line marks the threshold for left atrial enlargement (34 mL/m^2^). FDR-adjusted *q* < 0.001.

All 150 participants completed 6-month follow-up, and no deaths occurred. Forty-five patients (30.0%) experienced at least one documented readmission for heart-failure decompensation. Readmitted patients had markedly higher NT-proBNP, fibrinogen, CRP, and LAVI than patients who remained free from readmission, with all four comparisons remaining significant after FDR correction (*q* < 0.001). NLR did not differ significantly between groups. NYHA class III, hypertension, and atrial fibrillation were also substantially more frequent among readmitted patients (Table 1). The pronounced separation in CRP and LAVI distributions according to readmission status is illustrated in Figure 1 and Figure 2.

### 3.4. Correlation Structure and Functional Severity

**Figure 3:**
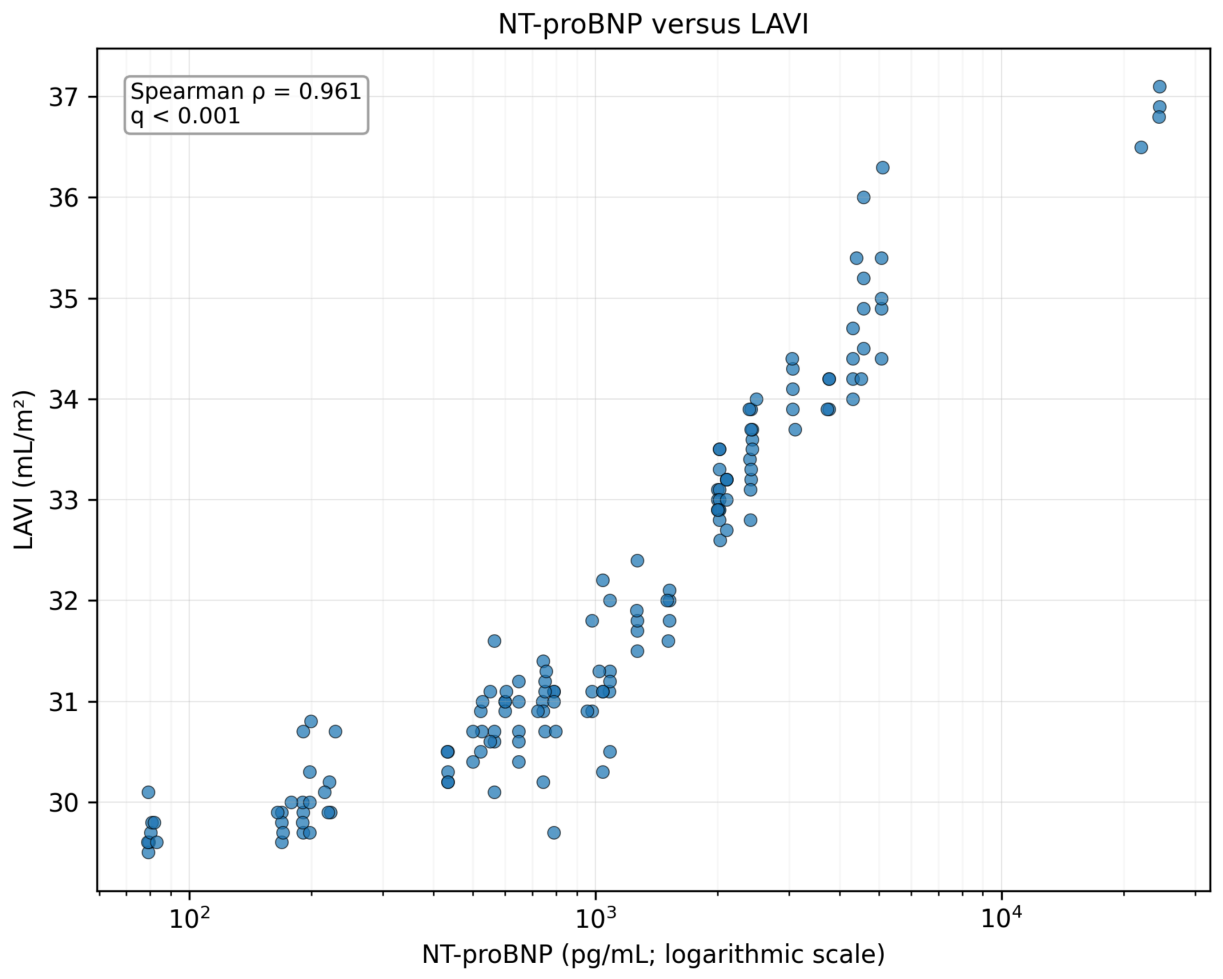
Scatterplot of NT-proBNP versus left atrial volume index (LAVI) in the 150 study participants. Each point represents one participant. NT-proBNP is shown on a logarithmic *x*-axis because of its right-skewed distribution. Spearman *ρ* = 0.961; FDR-adjusted *q* < 0.001. NT-proBNP, N-terminal pro-B-type natriuretic peptide.

**Figure 4:**
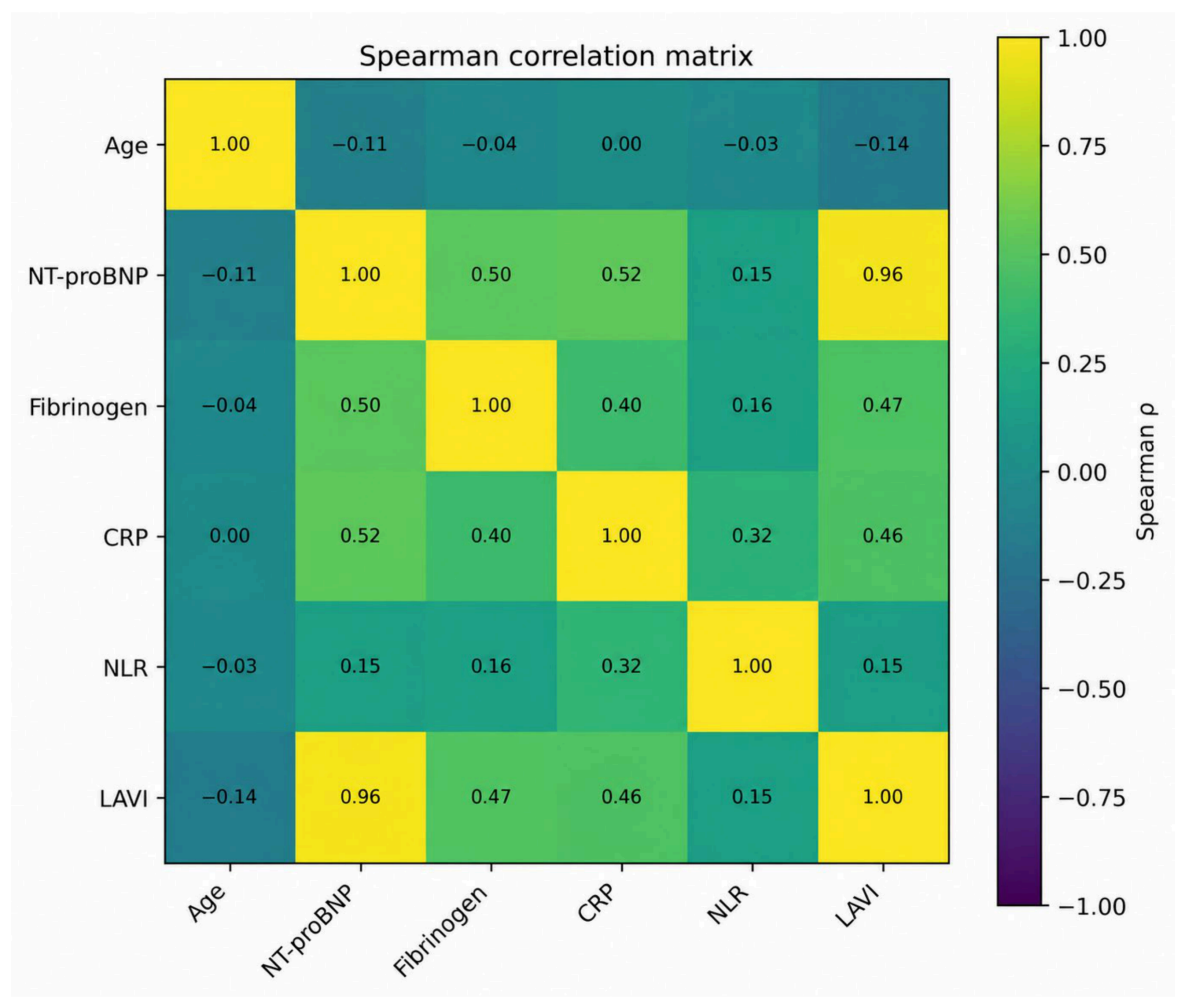
Spearman correlation matrix of continuous study variables. Cells show Spearman rank-correlation coefficients (ρ). The strongest association was between NT-proBNP and LAVI (*ρ* = 0.961; FDR-adjusted *q* < 0.001). CRP, C-reactive protein; LAVI, left atrial volume index; NLR, neutrophil-to-lymphocyte ratio; NT-proBNP, N-terminal pro-B-type natriuretic peptide.

The strongest continuous-variable association was observed between NT-proBNP and LAVI (Spearman *ρ* = 0.961, *q* < 0.001), indicating overlap between haemodynamic stress and left atrial structural remodelling in this cohort. The patient-level NT-proBNP–LAVI relationship is shown in Figure 3, with NT-proBNP displayed on a logarithmic axis because of its right-skewed distribution. NT-proBNP was also moderately correlated with CRP (*ρ* = 0.521, *q* < 0.001) and fibrinogen (*ρ* = 0.502, *q* < 0.001). Fibrinogen correlated with LAVI (*ρ* = 0.473), CRP correlated with LAVI (*ρ* = 0.457), and CRP correlated with NLR (*ρ* = 0.319; all *q* < 0.001). NLR was not significantly correlated with NT-proBNP after FDR adjustment. The complete correlation structure is presented in Figure 4.

Patients in NYHA class III had higher NT-proBNP (2389 (1042–4305) vs. 789 (229–2019) pg/mL), fibrinogen (380.2 ± 74.1 vs. 344.5 ± 58.1 mg/dL), CRP (5.00 (2.80–7.82) vs. 2.15 (1.10–3.40) mg/L), and LAVI (33.70 (31.10–34.30) vs. 31.10 (30.30–33.00) mL/m^2^) than patients in NYHA class II. Readmission occurred in 56.1% of NYHA class III patients compared with 20.2% of NYHA class II patients (*q* < 0.001).

### 3.5. Logistic Regression and Independent Predictors

In univariable logistic regression, readmission was associated with NYHA class III (OR 5.05, 95% CI 2.33–10.96), hypertension (OR 3.00, 95% CI 1.46–6.17), atrial fibrillation (OR 20.63, 95% CI 7.49–56.76), each doubling of NT-proBNP (OR 3.43, 95% CI 2.21–5.32), each 50 mg/dL increase in fibrinogen (OR 3.97, 95% CI 2.42–6.51), each doubling of CRP (OR 17.20, 95% CI 6.62–44.66), and each 1 mL/m^2^ increase in LAVI (OR 2.47, 95% CI 1.84–3.31; all FDR *q* < 0.01). NLR, age, sex, obesity, smoking, chronic kidney disease, anemia, and unadjusted etiology were not statistically significant predictors.

**Figure 5:**
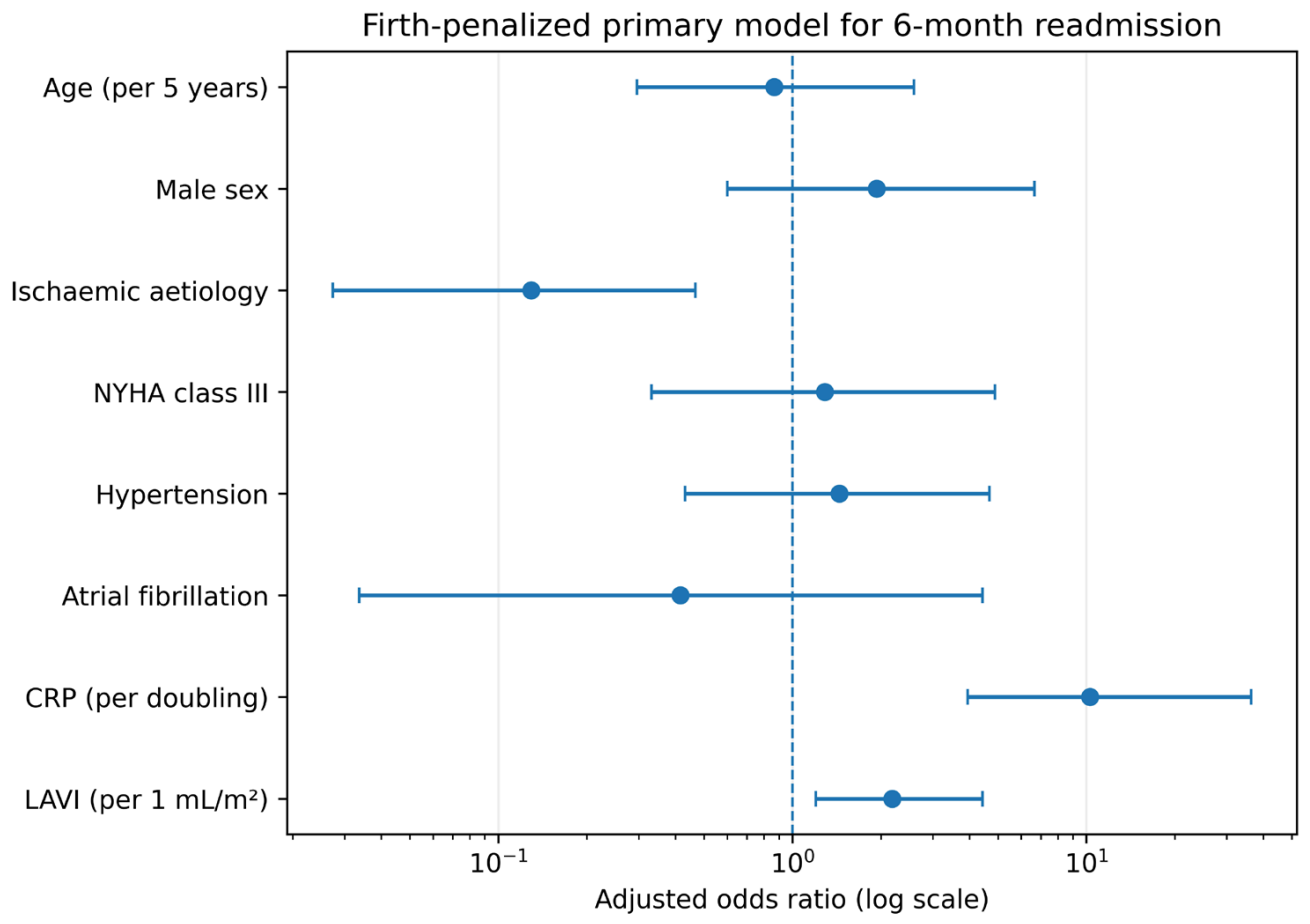
Adjusted odds ratios and 95% profile penalized-likelihood confidence intervals from the Firth-penalized primary model in which CRP and LAVI were added to the complete clinical model for 6-month readmission. The dashed vertical line denotes an odds ratio of 1. CRP was modelled per doubling and LAVI per 1 mL/m^2^ increase.

In the Firth-penalized clinical multivariable model, NYHA class III (adjusted OR 3.49, 95% CI 1.36–9.09; *p* = 0.010), hypertension (adjusted OR 2.68, 95% CI 1.12–6.59; *p* = 0.026), and atrial fibrillation (adjusted OR 11.36, 95% CI 4.21–34.32; *p* < 0.001) remained independently associated with readmission. In the primary penalized augmented model, CRP and LAVI were added without removing any clinical covariate. Each doubling of CRP was associated with a 10.29-fold increase in the odds of readmission (95% CI 3.94–36.33; *p* < 0.001), and each 1 mL/m^2^ increase in LAVI with a 2.18-fold increase (95% CI 1.20–4.43; *p* = 0.009). After addition of CRP and LAVI, hypertension (adjusted OR 1.44, 95% CI 0.43–4.68; *p* = 0.543), atrial fibrillation (adjusted OR 0.42, 95% CI 0.03–4.43; *p* = 0.470), and NYHA class III (adjusted OR 1.29, 95% CI 0.33–4.88; *p* = 0.708) were no longer independently significant (Table 2 and Figure 5). The inverse adjusted coefficient for ischaemic etiology (OR 0.13, 95% CI 0.03–0.47; *p* = 0.001) should be interpreted as a model-dependent suppression effect after conditioning on strongly prognostic clinical, inflammatory, and structural variables rather than as evidence of a protective biological effect.

The Firth-penalized clinical model had an apparent AUC of 0.855 and a bootstrap-corrected AUC of 0.826. Adding CRP and LAVI increased the apparent AUC to 0.963 and the bootstrap-corrected AUC to 0.943. The primary augmented model had an apparent Brier score of 0.072 and an optimism-corrected Brier score of 0.092. The apparent calibration intercept was −0.046 (95% CI −0.607 to 0.515), and the apparent calibration slope was 1.184 (95% CI 0.763–1.604); after 500-resample bootstrap correction for optimism, the calibration slope was approximately 0.785. The paired stratified-bootstrap estimate of the apparent AUC improvement was 0.107 (95% CI 0.054–0.167). Although Firth penalization reduced small-sample coefficient instability, these performance estimates remain internal validation of the derivation cohort only: only 45 readmission events were available, the calibration intervals were broad, and residual optimism and calibration uncertainty remain. An exploratory Firth-penalized model additionally containing fibrinogen achieved an apparent AUC of 0.985 and a bootstrap-corrected AUC of 0.970, but the additional model complexity relative to the number of events supported retaining the CRP + LAVI model as the principal model.

### 3.6. Discrimination and Threshold Performance

**Figure 6:**
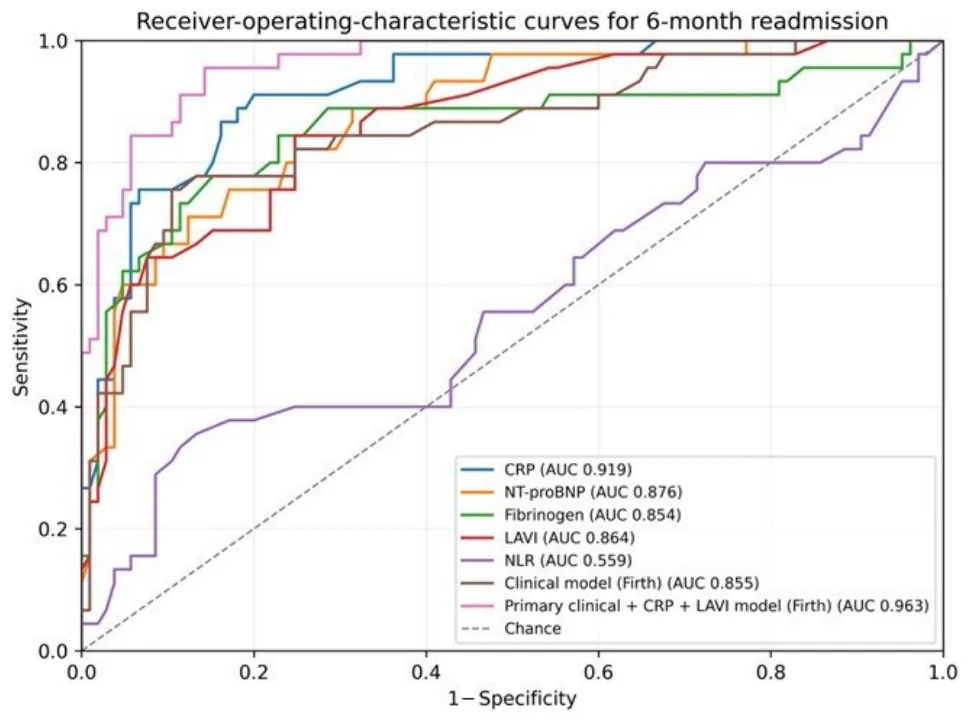
Receiver-operating-characteristic curves for individual biomarkers, the Firth-penalized clinical model, and the Firth-penalized primary model obtained by adding CRP and LAVI to the clinical model. The dashed diagonal denotes chance discrimination. CRP, C-reactive protein; LAVI, left atrial volume index; NLR, neutrophil-to-lymphocyte ratio; NT-proBNP, N-terminal pro-B-type natriuretic peptide.

Among individual markers, CRP showed the strongest discrimination for 6-month readmission (AUC 0.919, 95% CI 0.870–0.962), followed by NT-proBNP (AUC 0.876), LAVI (AUC 0.864), and fibrinogen (AUC 0.854). NLR demonstrated poor discrimination (AUC 0.559). The Firth-penalized primary model that augmented the complete clinical model with CRP and LAVI achieved an AUC of 0.963 (bootstrap 95% CI 0.933–0.985), an absolute improvement of 0.107 over the penalized clinical model (Figure 6 and Table 3). The paired stratified-bootstrap 95% CI for the AUC improvement was 0.054–0.167. The AUC differences between CRP and NT-proBNP, fibrinogen, or LAVI were not statistically significant, whereas CRP clearly outperformed NLR.

At the local NT-proBNP upper reference limit of >125 pg/mL, sensitivity for 6-month readmission was 100%, specificity 7.6%, PPV 31.7%, and NPV 100%. The very low specificity reflects the fact that this laboratory reference limit identifies abnormal natriuretic-peptide concentrations in established HFrEF rather than a prognostically optimized readmission threshold. At the prespecified CRP threshold > 5 mg/L, sensitivity was 62.2%, specificity 94.3%, PPV 82.4%, and NPV 85.3%. LAVI ≥ 34 mL/m^2^ had a sensitivity of 46.7%, specificity of 96.2%, PPV of 84.0%, and NPV of 80.8%. Fibrinogen > 498 mg/dL was observed in only four patients, all of whom were readmitted, yielding 100% specificity but only 8.9% sensitivity. The exploratory Youden thresholds were CRP 3.0 mg/L, LAVI 32.0 mL/m^2^, fibrinogen 390 mg/dL, and NT-proBNP 2102 pg/mL. The CRP 3.0 mg/L threshold achieved 91.1% sensitivity and 80.0% specificity, but all cohort-derived Youden thresholds are exploratory and require external validation before they can be considered for clinical application.

### 3.7. Calibration, Etiology-Specific Performance, and Sensitivity Analyses

**Figure 7:**
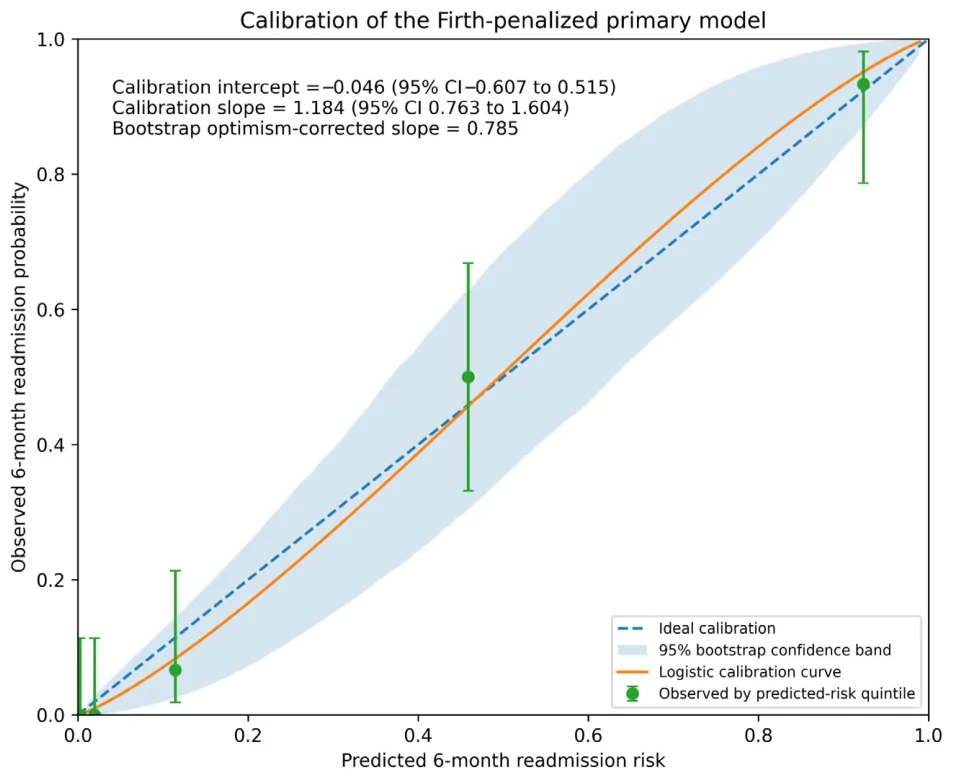
Calibration of the Firth-penalized primary clinical + CRP + LAVI model for 6-month readmission. The solid curve is the logistic recalibration curve and the shaded region its pointwise 95% bootstrap confidence band; the dashed diagonal denotes ideal calibration. Quintile points show observed readmission proportions at the corresponding mean predicted risks, with Wilson 95% confidence intervals. The apparent calibration intercept was −0.046 (95% CI −0.607 to 0.515), the apparent calibration slope was 1.184 (95% CI 0.763–1.604), and the 500-resample optimism-corrected slope was approximately 0.785. External validation is required.

For the Firth-penalized primary model, the mean predicted 6-month readmission risk was 30.4%, compared to an observed event rate of 30.0%. The apparent calibration intercept was −0.046 (95% CI −0.607 to 0.515), close to the ideal value of 0, and the apparent calibration slope was 1.184 (95% CI 0.763–1.604), with the interval including the ideal value of 1. The 500-resample optimism-corrected calibration slope was approximately 0.785. Figure 7 shows the logistic calibration curve with its pointwise 95% bootstrap confidence band, together with observed event proportions across quintiles of predicted risk and Wilson 95% CIs. The curve remained close to the ideal diagonal over much of the risk range. Because the model was developed and evaluated in the same cohort and only 45 events were observed, this remains apparent calibration; penalization and bootstrap internal validation reduce residual optimism, but external validation is required.

CRP remained strongly discriminative in both aetiological subgroups, with an AUC of 0.934 in idiopathic dilated cardiomyopathy and 0.940 in ischaemic cardiomyopathy. The corresponding LAVI AUCs were 0.835 and 0.914, respectively. No biomarker-by-etiology interaction remained statistically significant after FDR correction. A nominal fibrinogen interaction (*p* = 0.018) did not meet the multiplicity-adjusted threshold (*q* = 0.092). In the exploratory HC3-robust model of log2 NT-proBNP, LAVI was the dominant independent correlate (coefficient 0.822 per 1 mL/m^2^, *p* < 0.001), and the model explained 88.5% of the observed variance; this result largely reflected the strong NT-proBNP–LAVI relationship and should be interpreted as explanatory rather than causal.

## 4. Discussion

### 4.1. Principal Findings

The present study provides a multidimensional assessment of early readmission risk in clinically stable patients with HFrEF due to ischaemic or idiopathic dilated cardiomyopathy. Four principal findings emerged. First, systemic inflammatory activation, particularly higher CRP and fibrinogen, was strongly associated with 6-month heart-failure-related readmission. Second, LAVI provided powerful structural prognostic information. Third, and most importantly for model construction, adding CRP and LAVI to the complete conventional clinical model substantially improved discrimination while retaining all prespecified clinical predictors. CRP and LAVI remained independently associated with readmission in this augmented model, whereas the previously significant effects of hypertension and atrial fibrillation were attenuated. Fourth, NLR did not provide meaningful prognostic discrimination in this clinically stable cohort. All patients completed follow-up and no deaths occurred; consequently, the observed outcome burden represented non-fatal heart-failure morbidity rather than a composite of readmission and mortality.

### 4.2. Inflammatory Biomarkers and Residual Risk

Inflammation is increasingly recognized as a biologically relevant component of HFrEF, integrating myocardial injury, endothelial dysfunction, oxidative stress, congestion-related immune activation, and extracardiac comorbidity [7,8,9,10,11,12,13]. The marked separation in CRP concentrations between readmitted and non-readmitted patients, together with an individual AUC of 0.919 and a large adjusted effect per biomarker doubling, is consistent with previous evidence linking CRP to functional limitation, recurrent heart-failure hospitalization, and mortality [30,31,32,33,34,35,36,37,38,39]. The current analysis strengthens this interpretation because CRP remained strongly predictive after it was added to, rather than substituted for components of, a clinical model containing age, sex, etiology, NYHA class, hypertension, and atrial fibrillation.

Fibrinogen was also substantially higher among readmitted patients and showed good individual discrimination. This observation is biologically plausible because fibrinogen links acute-phase signalling to coagulation, blood viscosity, platelet activation, endothelial dysfunction, and atherothrombotic risk [42,43,44,45,46,47]. In the exploratory multimarker model, fibrinogen was added on top of the complete clinical + CRP + LAVI model. The fibrinogen result should be treated as hypothesis-generating rather than as evidence that a more complex multimarker model is ready for clinical application.

By contrast, NLR did not predict readmission in the present cohort, despite prior meta-analytic and cohort evidence supporting its prognostic relevance in acute and chronic HF [48,49,50,51,52,53,54,55,56]. Several explanations are possible. The exclusion of active inflammatory disease and recent decompensation may have narrowed the range of cellular inflammatory activation. NLR is also sensitive to infection, neuroendocrine stress, corticosteroid exposure, smoking, malignancy, and hematological conditions [54], and a single baseline measurement may not adequately reflect dynamic immune changes. The negative NLR result therefore does not invalidate its broader prognostic role, but suggests that CRP and fibrinogen were more informative in this selected, clinically stable population.

### 4.3. Left Atrial Remodelling, NT-proBNP, and Complementary Risk Information

LAVI is an integrative structural marker that reflects the cumulative consequences of chronically increased left ventricular filling pressures, diastolic burden, atrial stretch, and adverse remodelling. In this study, LAVI was markedly higher among readmitted patients, was associated with a large univariable effect, and remained independently predictive when added to the full clinical model together with CRP. The very strong correlation between LAVI and NT-proBNP (*ρ* = 0.961) suggests that both variables captured closely related haemodynamic and remodelling information. This finding also explains why simultaneous inclusion of both variables would have produced collinearity and unstable estimates. NT-proBNP remains a cornerstone of HF diagnosis and prognosis [6,14,15,16,17,18,19,20,21,22,23,24,25,26,27,28,29]; however, structural imaging may provide an intuitively interpretable measure of the chronic anatomical substrate that accompanies elevated wall stress.

Importantly, CRP correlated only moderately with NT-proBNP and LAVI, supporting partial biological independence between inflammatory activation and haemodynamic/structural stress. The key incremental comparison in the revised analysis is therefore between the same Firth-penalized clinical model before and after addition of CRP and LAVI: apparent AUC increased from 0.855 to 0.963, with a bootstrap-corrected AUC of 0.943 for the augmented model and a paired bootstrap AUC improvement of 0.107 (95% CI 0.054–0.167). Apparent calibration-in-the-large was close to ideal (intercept −0.046, 95% CI −0.607 to 0.515), and the apparent calibration slope was 1.184 (95% CI 0.763–1.604); however, the optimism-corrected slope of approximately 0.785, the broad confidence band around the calibration curve, and the limited number of events indicate residual calibration optimism. The attenuation of hypertension and atrial fibrillation after CRP and LAVI were introduced suggests that part of their unadjusted or clinical-model prognostic signal may overlap with inflammatory burden and chronic atrial/haemodynamic remodelling in this dataset. This should not be interpreted as evidence that these clinical factors are unimportant; rather, it illustrates how their independent coefficients change when more proximal markers of biological and structural risk are incorporated. Calibration, transportability, decision-curve performance, and incremental value against broader guideline-based risk models should be assessed in external cohorts before clinical use.

### 4.4. Clinical Implications for Early Risk Stratification

The findings are particularly relevant to early post-assessment risk stratification. CRP and LAVI are widely available, relatively inexpensive, and represent distinct pathophysiological domains. Notably, the observed readmissions occurred despite individualized, guideline-directed heart-failure therapy aligned with the contemporary ESC recommendations applicable during the study period, with the foundational treatment pillars prescribed and titrated to the maximally tolerated regimen whenever clinically feasible. A patient with elevated CRP and evidence of left atrial enlargement may therefore warrant closer surveillance, early reassessment of congestion and treatment tolerance, continued optimization of guideline-directed medical therapy, and more intensive evaluation of comorbidities. Such an approach is aligned with contemporary HF management, which emphasizes early comprehensive treatment and structured follow-up [1,2,3,4,5]. However, the present study was prognostic rather than interventional; it cannot demonstrate that management triggered by these markers reduces readmission.

The threshold analysis also illustrates the distinction between identifying a highly specific high-risk phenotype and screening broadly for vulnerability. CRP > 5 mg/L and LAVI ≥ 34 mL/m^2^ were highly specific but insufficiently sensitive, missing a substantial proportion of patients who were subsequently readmitted. The laboratory and echocardiographic reference limits should not be redefined on the basis of these data, and the exploratory, cohort-derived Youden cut-offs should be considered candidates for prospective external validation rather than proposed clinical standards.

### 4.5. Etiology and Model Interpretation

The prognostic performance of CRP was consistent across ischaemic and idiopathic dilated cardiomyopathy, and no biomarker-by-etiology interaction remained significant after FDR correction. This supports the potential relevance of inflammation across HFrEF aetiologies. Conversely, the inverse adjusted association between ischaemic etiology and readmission became more pronounced only after addition of strongly prognostic biomarkers and LAVI and was absent in the unadjusted analysis. The most plausible interpretation is statistical suppression caused by interrelationships among etiology, dyslipidaemia, clinical severity, and biomarker distributions. It should not be interpreted as evidence that ischaemic cardiomyopathy confers lower biological risk.

### 4.6. Strengths and Limitations

Strengths of this study include the balanced representation of two major HFrEF aetiologies, standardized laboratory reference limits, blinded echocardiographic assessment, complete 6-month readmission and vital-status ascertainment supported by medical documentation, absence of deaths and missing data, direct comparison of several accessible inflammatory markers, independent spreadsheet auditing of transformations and operating-characteristic calculations, Firth penalization of multivariable regression to reduce small-sample bias, and internal bootstrap validation rather than reliance on apparent discrimination alone. LAVI measurement was performed according to European transthoracic echocardiographic recommendations using dedicated apical four- and two-chamber views and the biplane modified Simpson method; three measurements were averaged in sinus rhythm, whereas five cardiac cycles were averaged in atrial fibrillation. The analysis also distinguished prespecified clinical thresholds from exploratory data-derived cut-offs and explicitly addressed collinearity between NT-proBNP and LAVI.

Several limitations require emphasis. The study was conducted at a single centre and included only 150 patients and 45 readmission events, limiting precision, interaction power, and the number of regression parameters that could be estimated reliably. This limitation is especially relevant to the primary model, which contains eight predictors, and to the nine-predictor exploratory multimarker model. Firth bias-reduced penalized regression was therefore used to mitigate small-sample coefficient bias and instability, but penalization does not replace external validation. The regression models were established after the analytical database had been constituted; accordingly, the present work should be regarded as model derivation and hypothesis generation. Although bootstrap internal validation provides an estimate of optimism within the present dataset, it cannot establish transportability to other populations or centres. The marked attenuation and direction change in the atrial-fibrillation coefficient after addition of CRP and LAVI should be interpreted as model-dependent rather than as evidence of a protective effect. The observational design precludes causal inference. A specific limitation concerns time-to-event analysis. Follow-up began separately for each patient on the date of enrolment and continued for 6 months, but the database retained only whether at least one heart-failure-related readmission occurred during that interval; it did not retain the exact date of readmission. Accordingly, the present analysis can estimate the probability of readmission within 6 months but cannot characterize event-free survival, the timing or hazard of readmission during follow-up, or temporal differences in risk between patients. Recurrent-hospitalization counts were also unavailable, precluding recurrent-event modelling. In addition, the database did not capture diabetes mellitus, the number or burden of heart-failure hospitalizations before the prespecified 6-month stability window, the duration of heart failure, or treatment with intracardiac devices such as implantable cardioverter-defibrillators or cardiac resynchronization therapy; these factors may influence prognosis and represent potential sources of residual confounding. All patients received guideline-directed medical therapy according to the ESC recommendations applicable during the study period, with the foundational treatment pillars used whenever tolerated and treatment adjusted individually to the maximally tolerated regimen. However, the analytical database did not capture each individual medication and dose for every participant; therefore, medication-specific exposure, achieved target-dose proportions, and dose-response relationships could not be analyzed. Statistically meaningful analysis of pharmacological treatment would require a substantially larger cohort because contemporary HFrEF therapy comprises four foundational treatment pillars, multiple drug options within several classes, and multiple dose and titration states, creating a high-dimensional treatment-exposure space relative to the number of outcome events in the present study. Although HFrEF eligibility required LVEF < 40%, patient-level LVEF values were not reported as a continuous variable in the analytical results; therefore, the distribution and severity of systolic dysfunction could not be described or incorporated as a graded covariate. Other unavailable variables included renal-function values, blood pressure, socioeconomic factors, adherence, and other established determinants of readmission, leaving residual confounding. No deaths occurred during follow-up, which removed death as a competing event but precluded assessment of mortality or composite clinical outcomes. Finally, biomarkers and LAVI were measured only once; temporal trajectories and treatment-related changes therefore require evaluation in future studies.

Future multicentre studies should externally and prospectively validate the Firth-penalized clinical model augmented with CRP and LAVI in a substantially larger and more heterogeneous independent HFrEF cohort, incorporate diabetes mellitus, prior heart-failure hospitalization burden, heart-failure duration, intracardiac device therapy, continuous LVEF, renal function, blood pressure, and detailed contemporary pharmacological treatment data, assess serial biomarker and echocardiographic changes, and evaluate clinical usefulness using calibration-in-the-large, decision-curve analysis, and net-benefit methods. Larger cohorts with substantially more outcome events are particularly necessary for treatment-effect analyses because the four foundational HFrEF drug pillars, multiple agents within drug classes, and multiple dose/titration levels generate many clinically relevant exposure combinations. Such external validation is necessary to assess transportability, recalibration requirements, and reproducibility before any clinical implementation of the proposed model is considered. Model updating or derivation of a clinically deployable risk score should only follow successful external validation.

## 5. Conclusions

In patients with HFrEF due to ischaemic or idiopathic dilated cardiomyopathy, systemic inflammatory activation and left atrial remodelling were strongly associated with heart-failure-related readmission within 6 months. When CRP and LAVI were added to the complete clinical model rather than replacing hypertension and atrial fibrillation, both remained independent predictors in Firth bias-reduced penalized regression. The augmented model achieved an apparent AUC of 0.963 and a bootstrap-corrected AUC of 0.943, compared to 0.855 and 0.826 for the clinical model, respectively. Apparent calibration-in-the-large was close to ideal (intercept −0.046, 95% CI −0.607 to 0.515), while the apparent calibration slope was 1.184 (95% CI 0.763–1.604) and the optimism-corrected slope was approximately 0.785. These remain internally validated derivation results based on only 45 readmission events and some residual optimism in calibration.

These findings support a multimodal approach to early risk stratification in which accessible inflammatory and structural markers complement established clinical information. Because the models were established after constitution of the analytical database, the observed effect sizes and model performance should be regarded as internally validated, hypothesis-generating derivation results. Bootstrap validation cannot substitute for independent external validation, particularly given the limited number of adverse events. Validation in a larger external HFrEF cohort, together with model recalibration and prospective assessment of clinical utility, is required before clinical implementation or use of the augmented model to direct individualized follow-up or treatment strategies can be considered.

## Figures and Tables

**Table 1:** Clinical, laboratory, and echocardiographic characteristics according to 6-month readmission status.

Variable	Overall	No Readmission (*n* = 105)	Readmission (*n* = 45)	*p*-Value	FDR *q*-Value
Age, years	60.7 ± 3.0	60.8 ± 3.1	60.5 ± 2.8	0.520	0.618
NT-proBNP, pg/mL	1043 (526–2404)	648 (229–1267)	3058 (2019–4567)	<0.001	<0.001
Fibrinogen, mg/dL	354.3 ± 64.6	331.3 ± 52.3	407.8 ± 59.0	<0.001	<0.001
CRP, mg/L	2.80 (1.62–4.58)	2.12 (1.00–2.84)	5.60 (3.86–7.82)	<0.001	<0.001
NLR	2.36 (1.83–2.93)	2.32 (1.79–2.85)	2.40 (1.90–3.50)	0.253	0.370
LAVI, mL/m^2^	31.30 (30.52–33.38)	30.90 (30.20–31.90)	33.90 (32.70–34.50)	<0.001	<0.001
Male sex	91 (60.7%)	59 (56.2%)	32 (71.1%)	0.086	0.149
NYHA class III	41 (27.3%)	18 (17.1%)	23 (51.1%)	<0.001	<0.001
Obesity	42 (28.0%)	29 (27.6%)	13 (28.9%)	0.874	0.874
Current smoking	78 (52.0%)	57 (54.3%)	21 (46.7%)	0.392	0.497
Hypertension	62 (41.3%)	35 (33.3%)	27 (60.0%)	0.002	0.005
Dyslipidemia	101 (67.3%)	75 (71.4%)	26 (57.8%)	0.102	0.162
CKD stage ≥ 3	19 (12.7%)	13 (12.4%)	6 (13.3%)	0.872	0.874
Anemia	7 (4.7%)	6 (5.7%)	1 (2.2%)	0.675	0.754
Atrial fibrillation	31 (20.7%)	6 (5.7%)	25 (55.6%)	<0.001	<0.001
CRP > 5 mg/L	34 (22.7%)	6 (5.7%)	28 (62.2%)	<0.001	<0.001
Fibrinogen < 238 mg/dL	4 (2.7%)	4 (3.8%)	0 (0.0%)	0.317	0.430
Fibrinogen > 498 mg/dL	4 (2.7%)	0 (0.0%)	4 (8.9%)	0.007	0.014
LAVI ≥ 34 mL/m^2^	25 (16.7%)	4 (3.8%)	21 (46.7%)	<0.001	<0.001

Data are mean ± SD, median [IQR], or *n* (%). Continuous comparisons used Welch’s *t*-test or the Mann–Whitney U test, and categorical comparisons used Pearson’s chi-square or Fisher’s exact test, as appropriate. FDR, false discovery rate; LAVI, left atrial volume index; NLR, neutrophil-to-lymphocyte ratio; NT-proBNP, N-terminal pro-B-type natriuretic peptide.

**Table 2:** Primary Firth-penalized multivariable logistic regression model: clinical predictors augmented with CRP and LAVI for 6-month readmission.

Predictor	Adjusted OR	95% CI	*p*-Value
Age (per 5 years)	0.87	0.30–2.59	0.792
Male sex	1.93	0.60–6.65	0.271
Ischemic etiology	0.13	0.03–0.47	0.001
NYHA class III	1.29	0.33–4.88	0.708
Hypertension	1.44	0.43–4.68	0.543
Atrial fibrillation	0.42	0.03–4.43	0.470
CRP (per doubling)	10.29	3.94–36.33	<0.001
LAVI (per 1 mL/m^2^)	2.18	1.20–4.43	0.009

Age is expressed per 5-year increase, CRP per doubling, and LAVI per 1 mL/m^2^ increase. The primary model retained age, sex, cardiomyopathy etiology, NYHA class III, hypertension, and atrial fibrillation and added CRP and LAVI. Adjusted ORs are from Firth bias-reduced penalized logistic regression; 95% CIs are profile penalized-likelihood intervals and *p*-values are from penalized likelihood-ratio tests. CI, confidence interval; CRP, C-reactive protein; LAVI, left atrial volume index; OR, odds ratio.

**Table 3:** Discrimination and data-derived operating characteristics for 6-month readmission.

Marker/Model	AUC	95% CI	Youden Cut-Off	Sensitivity	Specificity	PPV	NPV
NT-proBNP	0.876	0.813–0.931	2102	71.1%	87.6%	71.1%	87.6%
Fibrinogen	0.854	0.767–0.926	390	77.8%	84.8%	68.6%	89.9%
CRP	0.919	0.870–0.962	3	91.1%	80.0%	66.1%	95.5%
NLR	0.559	0.452–0.673	3.060	35.6%	86.7%	53.3%	75.8%
LAVI	0.864	0.794–0.922	32	84.4%	75.2%	59.4%	91.9%
Clinical model	0.855	0.783–0.918	0.337	75.6%	89.5%	75.6%	89.5%
Primary clinical + CRP + LAVI model	0.963	0.933–0.985	0.237	95.6%	85.7%	74.1%	97.8%
Exploratory multimarker model	0.985	0.968–0.996	0.309	95.6%	91.4%	82.7%	98.0%

AUC confidence intervals were obtained by stratified bootstrap. Model probabilities were generated from Firth-penalized logistic regression. Youden cut-offs were optimized in the same cohort, are exploratory, and require external validation before any clinical use. The bootstrap-corrected AUCs were 0.826 for the clinical model, 0.943 for the primary clinical + CRP + LAVI model, and 0.970 for the exploratory multimarker model. AUC, area under the curve; NPV, negative predictive value; PPV, positive predictive value.

## Data Availability

The original contributions presented in this study are included in the article. Further inquiries can be directed to the corresponding author.

## Abbreviations

The following abbreviations are used in this manuscript:

AF	atrial fibrillation
AIC	Akaike information criterion
AUC	area under the receiver-operating-characteristic curve
BIC	Bayesian information criterion
BMI	body mass index
CI	confidence interval
CKD	chronic kidney disease
CRP	C-reactive protein
DCM	dilated cardiomyopathy
ECLIA	electrochemiluminescence immunoassay
ELISA	enzyme-linked immunosorbent assay
FDR	false discovery rate
HC3	heteroskedasticity-consistent type 3
HF	heart failure
HFrEF	heart failure with reduced ejection fraction
IQR	interquartile range
LAVI	left atrial volume index
LVEF	left ventricular ejection fraction
NLR	neutrophil-to-lymphocyte ratio
NPV	negative predictive value
NT-proBNP	N-terminal pro-B-type natriuretic peptide
NYHA	New York Heart Association
OR	odds ratio
PPV	positive predictive value
ROC	receiver-operating-characteristic
SD	standard deviation
